# The Correlation between Cerebral Blood Flow Measured by Bedside Xenon-CT and Brain Chemistry Monitored by Microdialysis in the Acute Phase following Subarachnoid Hemorrhage

**DOI:** 10.3389/fneur.2017.00369

**Published:** 2017-08-02

**Authors:** Elham Rostami, Henrik Engquist, Timothy Howells, Elisabeth Ronne-Engström, Pelle Nilsson, Lars Tomas Hillered, Anders Lewén, Per Enblad

**Affiliations:** ^1^Section of Neurosurgery, Department of Neuroscience, Uppsala University, Uppsala, Sweden; ^2^Anesthesiology and Intensive Care, Department of Surgical Sciences, Uppsala University, Uppsala, Sweden

**Keywords:** cerebral blood flow, microdialysis, lactate, Xenon-CT, subarachnoid hemorrhage

## Abstract

Cerebral microdialysis (MD) may be used in patients suffering from subarachnoid hemorrhage (SAH) to detect focal cerebral ischemia. The cerebral MD catheter is usually placed in the right frontal lobe and monitors the area surrounding the catheter. This generates the concern that a fall in cerebral blood flow (CBF) and ischemic events distant to the catheter may not be detected. We aimed to investigate if there is a difference in the association between the MD parameters and CBF measured around the MD catheter compared to global cortical CBF and to CBF in the vascular territories following SAH in the early acute phase. MD catheter was placed in the right frontal lobe of 30 SAH patients, and interstitial glucose, lactate, pyruvate, glycerol, and lactate/pyruvate ratio were measured hourly. CBF measurements were performed during day 0–3 after SAH. Global cortical CBF correlated strongly with CBF around the microdialysis catheter (CBF-MD) (*r* = 0.911, *p* ≤ 0.001). This was also the case for the anterior, middle, and posterior vascular territories in the right hemisphere. A significant negative correlation was seen between lactate and CBF-MD (*r* = −0.468, *p* = 0.009). The same relationship was observed between lactate and CBF in anterior vascular territory but not in the middle and posterior vascular territories. In conclusion, global CBF 0–3 days after severe SAH correlated strongly with CBF-MD. High lactate level was associated with low global CBF and low regional CBF in the right anterior vascular territory, when the MD catheter was placed in the right frontal lobe.

## Introduction

Today multimodal monitoring is a part of the neurointensive care (NIC) management of patients suffering severe subarachnoid hemorrhage (SAH) (1). Microdialysis (MD) of the extracellular fluid may be used to monitor the metabolic state of the tissue in order to detect secondary injuries such as ischemia (2, 3). Cerebral ischemia is a feared complication which occurs in 20–30% of patients suffering from SAH and increases the morbidity and mortality (4). Monitoring cerebral metabolites and cerebral blood flow (CBF) provides vital information on tissue at risk of developing ischemia. However, MD is a focal technique that measures a small region of the brain tissue, and it is recommended that, if possible, the MD catheter should be placed in the vascular territory at risk (5). At our department, the MD catheter is routinely placed in right frontal lobe. This is based on the assumption that both the middle cerebral artery (MCA) and the anterior cerebral artery (ACA) territories will be monitored. Bedside Xenon-CT is used routinely in our NIC unit in order to assess the regional CBF in patients following SAH (6–8). In a previous Xenon-CT study including 64 SAH patients, we could not find any correlation between regional CBF and aneurysm location (7).

Earlier studies in SAH patients using positron emission tomography simultaneously with MD have shown increased levels of energy metabolites (9) and glutamate (10) under conditions with low CBF.

The objective of the current study, using bedside Xenon-CT, was to investigate if there is a difference in the association between the MD parameters and CBF measured around the MD catheter compared to global cortical CBF and to CBF in the vascular territories during the early acute phase of SAH.

## Materials and Methods

### Study Population and Study Design

Thirty patients with SAH who were admitted to the NIC unit, Section of Neurosurgery, Uppsala University Hospital, between October 2012 and May 2015 were included in the study.

The inclusion criteria were patients who underwent a Xenon-CT at day 0–3 after onset of SAH and received a MD catheter at admission. These patients needed to be mechanically ventilated for the Xenon-CT and in need of a ventriculostomy for simultaneous insertion of a MD catheter. Patients with a preexisting neurological deficit, an SAH resulting from trauma, or arteriovenous malformation were excluded. The SAH was verified by CT scanning and the aneurysm was visualized by a CT angiography and/or digital subtraction angiography (6).

### Neurointensive Care

The standardized protocol at our NIC unit, which is well described previously (6, 11), is based on intensive physiological monitoring and aggressive therapy of any derangement to avoid or minimize secondary brain injury. Unconscious patients are mechanically ventilated and receive a ventriculostomy. If ICP is above 20 mmHg, the drainage system is opened and cerebrospinal fluid drained against a pressure level of 15 mmHg. Hypotension is treated first with albumin 20% and crystalloid solutions, and with Dobutamine (Algol Pharma AB, Kista, Sweden) if needed. The goal is to keep CPP above 60 mmHg. Identified aneurysms are treated early by endovascular coiling or surgical clipping. All patients receive nimodipine (Nimotop^®^, Bayer AB, Solna, Sweden).

### CBF Measurements

At our department, bedside Xenon-CT has been introduced as a routine and is performed on patients with SAH and mechanically ventilated at day 0–3, day 4–7 and after 7 days after admission as far as the necessary resources are available (6). This time point is used since delayed cerebral ischemia (DCI) is rarely seen before day 3 following onset of SAH (12, 13). The principal of CBF measurements using Xenon-CT has been previously described by Yonas et al. (14–16), and the procedure used at our department has also been previously described (6). Briefly, a concentration of 28% of stable Xenon is administered to the patients breathing circuit for about 4 min using the Enhancer 3000 and the specially developed computer software (Diversified Diagnostic Products Inc., Houston, TX, USA). During the Xenon inhalation, eight CT scans at four different levels with 10-mm spacing are obtained by the CereTom^®^ (Neurologica, Boston, MA, USA). The first CT-scan is adjusted using the scout view in order to avoid inclusion of coil artifacts. The computer software synchronizes the Xenon delivery and the CT scans. The resulting radiologic tissue enhancement of the Xenon wash-in enables CBF (ml/100 g/min) to be calculated and plotted as colored maps.

**Figure 1:**
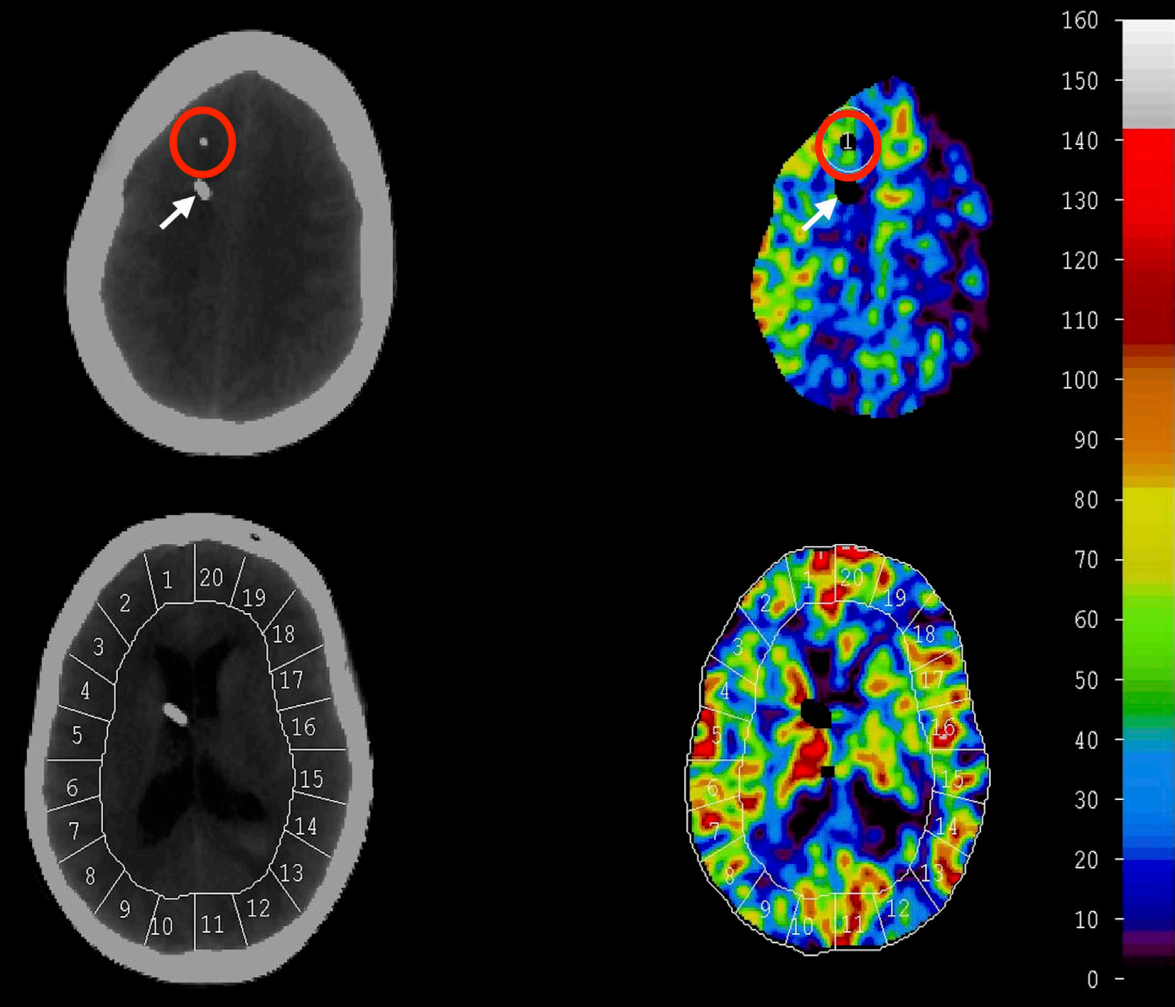
Xenon-CT scans at different levels obtained by bedside mobile CT-scanner. Conventional CT images are obtained for evaluation and identification of microdialysis (MD) catheter. Following Xenon delivery tissue enhancement of the Xenon wash-in enabled cerebral blood flow (CBF) (ml/100 g/min) to be calculated and plotted as colored maps. Scale of CBF is ml/100 g/min and is given to the right. Twenty cortical ROIs were used for CBF calculation and regional vascular territory was identified (anterior cerebral artery 1–2, 19–20, medial cerebral artery 3–8, 13–18, posterior cerebral artery 9–10, 11–12). CBF around the MD catheter was calculated by drawing an ROI manually around the catheter (circle in red). White arrow indicates EVD.

Mean blood flow in each of 20 evenly distributed cortical regions (ROIs) was calculated for each level, and the global CBF is given as a mean of all four levels. The vascular territories were analyzed as following: ACA—ROI 1–2 (right) and 19–20 (left), MCA—ROI 3–8 (right) and 13–18 (left), and posterior cerebral artery (PCA)—ROI 9–10 (right) and 11–12 (left) (Figure 1). The tip of the MD catheter was identified on the structural CT scans and an ROI was drawn manually (diameter = 3 cm) for the corresponding area around the MD catheter on the CBF scans. Territories with CT-defined hematoma or artifacts were noted and excluded.

### Cerebral MD

The cerebral MD technique in NIC has previously been extensively used and described (2, 9). The intracerebral MD catheter is placed in the right frontal lobe cortex through a separate burr hole, anterior to the ventricular drain. For intracerebral MD monitoring, a 70 brain MD catheter is used (M Dialysis AB, Stockholm, Sweden) with a membrane length of 10 mm and a membrane cutoff of 20,000 Da. The catheters are perfused with artificial CSF (NaCl 147 mmol/l, KCl 2.7 mmol/l, CaCl_2_ 1.2 mmol/l, MgCl_2_ 0.85 mmol/l) (perfusion fluid CNS; M Dialysis AB).

The perfusion rate was measured as 0.3 µl/min using a microinjection pump (CMA-106, M Dialysis AB). MD urea was monitored to validate catheter performance (17). The MD samples were collected on an hourly basis. For the correlation analysis, the MD sample was collected at the end of the Xenon-CT exanimation. Interstitial glucose, lactate, pyruvate, glutamate, glycerol, and urea were analyzed enzymatically using a CMA 600 analyzer or ISCUS Clinical Microdialysis Analyzer (M Dialysis AB).

### Statistical Analysis

All analyses were performed using SPSS Statistics for Macintosh, Version 23.0 (IBM^®^, Armonk, NY, USA). In order to assess the normality of the data set, the skewness and kurtosis of the distribution were analyzed. Since the parameters were not normally distributed, Spearman’s correlation was used. Bonferroni correction was performed for multiple comparisons. Results are expressed as mean ± SD and range within brackets. A *p* value <0.05 was considered statistically significant.

### Ethics

The Uppsala University Regional Ethics Review Board for clinical research granted permission to undertake the study. Written informed consent was obtained from all patients or their proxy for study participation. The study was also approved by the local Radiation Safety Authority.

## Results

### Demography and Clinical Data

Thirty patients with severe SAH were included, 5 males and 25 females. Demographics and clinical data including the distribution of aneurysm location are given in Table 1. The physiological parameters were stable during CBF measurements, and baseline values are shown in Table 2.

**Table 1:** Demographics and clinical data including the distribution of aneurysm location.

Patient characteristics	*n* (%)
**Sex**	
Female	25 (83)
Male	5 (16)
**Age (year)**	58.9 (28–84)
Hunt and Hess at admission	
H&H I–II	6 (20)
H&H III	8 (26)
H&H IV–V	16 (53)
**Fisher grade**	
1–2	0 (0)
3	7 (23)
4	23 (76)
**Aneurysm location**	
AComA	9 (30)
ICA	4 (13)
PComA	5 (16)
MCA	6 (20)
AChA	1 (3)
PCA	1 (3)
BA	1 (3)
PICA	2 (6)
Unknown	1 (3)
**Treatment**	
Clip	4 (13)
Coil	25 (83)

*Age is given as mean and (range)*.

*AcomA, anterior communicating artery; ICA, internal carotid artery; PComA, posterior communicating artery; MCA, middle cerebral artery; AChA, anterior choroidal artery; PCA, posterior cerebral artery; BA, basilar artery; PICA, posterior inferior cerebellar artery*.

**Table 2:** Physiological parameters before and after the Xenon-CT measurements.

	Before	After
PaO_2_ (kPa)	13.3 ± 2.8	13.6 ± 3
PaCO_2_ (kPa)	5.1 ± 0.4	5.2 ± 0.5
FIO_2_ (%)	39.3 ± 10.3	39.6 ± 10.1
MAP (mmHg)	94.1 ± 14.1	90.5 ± 11.8
ICP (mmHg)	17 ± 5	17.5 ± 4.7
CPP (mmHg)	77.6 ± 15.5	76.9 ± 13.1

*Values are given as mean ± SD*.

*PaO_2_, arterial partial pressure of oxygen; PaCO_2_, arterial partial pressure of carbon dioxide; FIO_2_, fraction of inspired oxygen; MAP, mean arterial blood pressure; ICP, intracranial pressure; CPP, cerebral perfusion pressure*.

### CBF Measurements

The mean cortical global CBF for all patients was 33.3 ± 13.5 ml/100 g/min (13.7–73.8) and CBF-MD was 30.3 ± 12.5 ml/100 g/min (11.0–56.9). There was a significant positive correlation between the global CBF and CBF-MD (*r* = 0.911, *p* ≤ 0.001).

**Figure 2:**
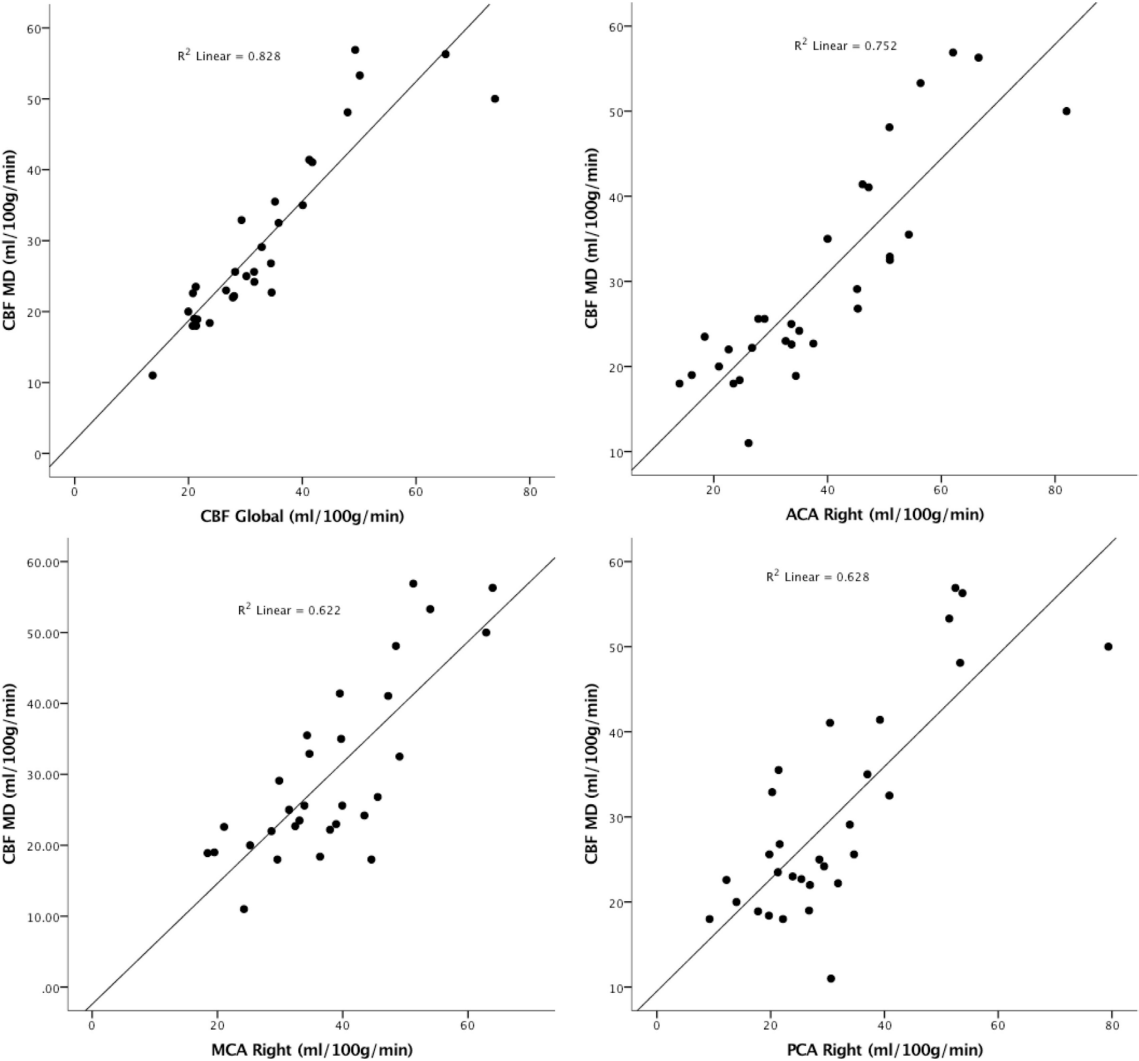
The correlation between global cerebral blood flow (CBF) and CBF around the microdialysis catheter (CBF-MD) and different vascular territories in the right hemisphere.

The correlation between CBF around the MD catheter and different vascular territories of the right hemisphere was calculated and showed to be significant (*p* ≤ 0.001) (Figure 2). Stronger correlation was seen between CBF in the ACA territory and MD.

### MD Results

There were no complications associated with MD catheter insertion. The MD data corresponding to the time of CBF measurements are shown in Table 3. There was a huge variation between the patients, in particular in glycerol and glutamate.

**Table 3:** Microdialysis data for all patients (*n* = 30) at the time of cerebral blood flow measurements.

	Glucose (mmol/l)	Lactate (mmol/l)	Pyruvate (μmol/l)	Glycerol (μmol/l)	Glutamate (μmol/l)	L/P ratio
Mean ± SD	2.3 ± 1.1	3.9 ± 2	143.6 ± 46.8	147.5 ± 149.3	32.2 ± 57.7	27.6 ± 11.8
Range	0.5 – 5.3	1.4 – 10.4	66.7 – 249.4	18 – 577	0.2 – 170	15.2 – 71.2

Pyruvate showed a strong positive correlation with lactate which remained significant after Bonferroni correction (*r* = 0.738, *p* ≤ 0.001) and glucose (*r* = 0.496, *p* = 0.006), but the correlation with glucose did not pass the Bonferroni correction level. Glutamate showed the strongest correlation with lactate although not significant (*r* = 0.375, *p* = 0.049).

### CBF and MD Correlation

Microdialysis parameters obtained at the time of Xenon-CT were compared to global cortical CBF and CBF-MD to investigate if there was an association between the CBF and the interstitial chemistry.

A significant negative correlation could be seen between lactate and CBF-MD (*r* = −0.468, *p* = 0.009). Lactate also negatively correlated with global CBF (*r* = −0.408, *p* = 0.025) but this did not remain significant following Bonferroni correction. There was a weak negative and non-significant correlation between L/P ratio and CBF-MD (*r* = −0.364, *p* = 0.048) and global CBF (*r* = −0.329, *p* = 0.075). No significant correlation could be found between CBF and glucose, pyruvate, glycerol, and glutamate.

**Figure 3:**
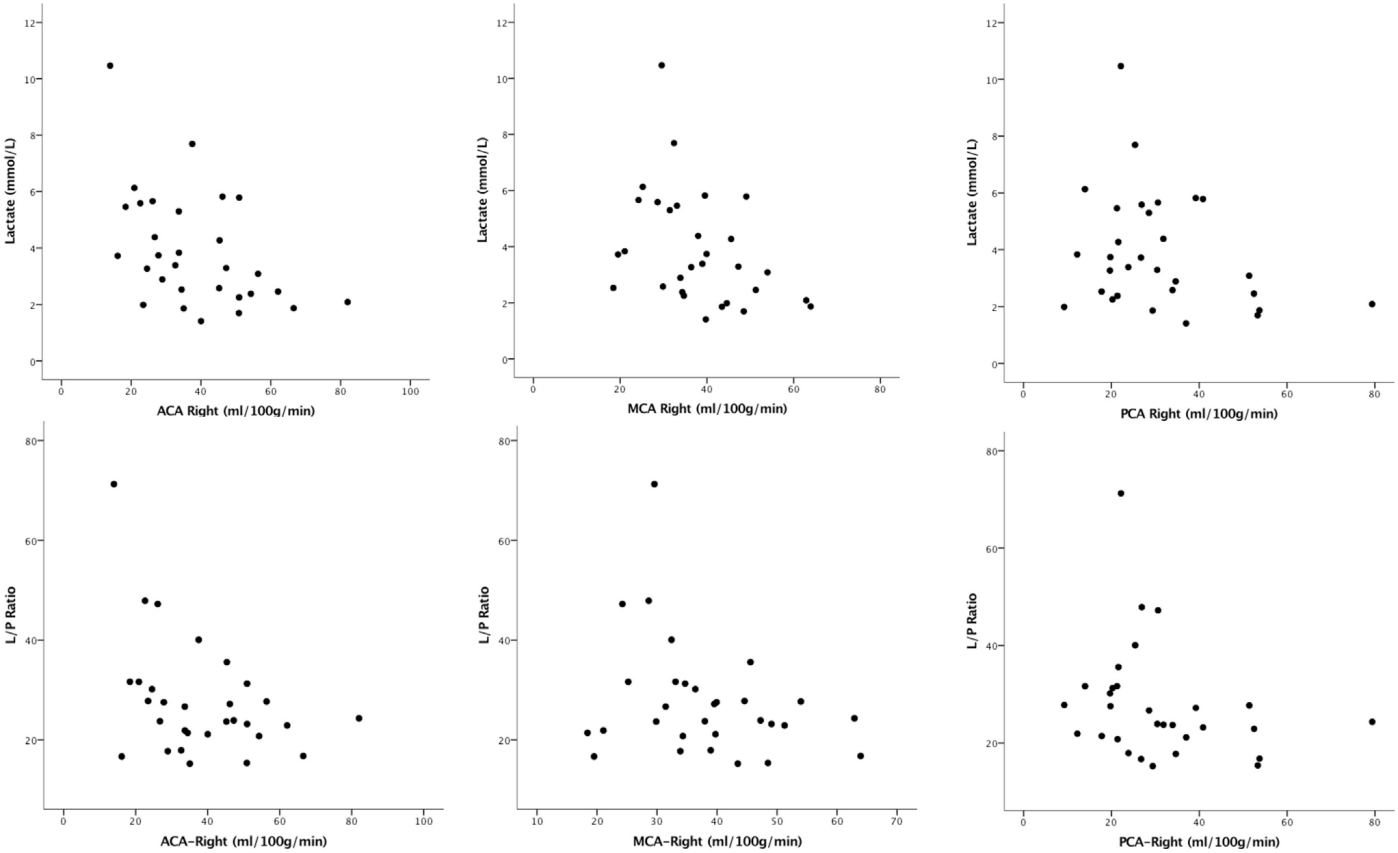
The correlation between cerebral blood flow (CBF) in each vascular territory of the right hemisphere and microdialysis lactate/pyruvate ratio and lactate is shown. Lactate showed a significant correlation with CBF in anterior cerebral artery (ACA) territory in the right hemisphere.

The association between CBF in each vascular territory in the right hemisphere and MD parameters was investigated (Table 4). Lactate showed a significant negative correlation with CBF in ACA in the right hemisphere. This correlation was weaker and non-significant for CBF in PCA territory (Figure 3). After Bonferroni correction, only lactate correlated significantly with CBF in ACA territory.

**Table 4:** Correlation between cerebral blood flow (CBF) measurements in different vascular territories of right hemisphere and microdialysis parameters.

	Right ACA—CBF		Right MCA—CBF		Right PCA—CBF	
	*r*	*p-*Value	*r*	*p-*Value	*r*	*p-*Value
Glucose (mmol/l)	0.004	0.982	0.161	0.402	0.149	0.441
Pyruvate (μmol/l)	−0.363	0.049	−0.355	0.054	−0.120	0.526
Glycerol (μmol/l)	−0.026	0.897	0.007	0.971	0.119	0.545
Glutamate (μmol/l)	−0.324	0.093	−0.245	0.141	−0.067	0.736
Lactate (mmol/l)	−0.482	0.007^a^	−0.460	0.010	−0.242	0.198
L/P ratio	−0.354	0.055	−0.229	0.223	−0.348	0.060

*The r-value is the Spearman correlation coefficient*.

*CBF, cerebral blood flow; L/P ratio, lactate/pyruvate ratio; ACA, anterior cerebral artery; MCA, middle cerebral artery and PCA, posterior cerebral artery*.

*^a^Significant correlation after Bonferroni correction for multiple comparisons*.

## Discussion

In this study, we found that bedside monitoring of CBF using Xenon-CT in combination with MD in patients with SAH was feasible and safe. Placement of the MD catheter in the right frontal lobe following SAH showed a strong negative correlation between lactate and regional CBF in the anterior vascular territory but not in the middle and the posterior vascular territories. Few studies have combined CBF measurements with MD parameters in SAH patients (9, 10, 18). Using PET, Enblad et al. found that lactate, L/P ratio, and glutamate had the highest sensitivity for detecting ischemia in the area of the MD catheter (9). Also, Sarrafzadeh et al. found highest sensitivity for lactate and glutamate to detect ischemia using PET (10, 18).

High lactate levels have been reported to be associated with ischemia both in SAH patients and patients with head injury (19, 20). However, high lactate levels may also indicate hyperglycolysis (21, 22), explaining its rather low specificity as a biomarker of ischemia (9). Consequently, additional parameters such as L/P-ratio, pyruvate, and CBF should be evaluated to distinguish between ischemia, hyperglycolysis, and mitochondrial dysfunction (5, 23–25). In the current study, there was a significant negative correlation between CBF and lactate. However, the L/P ratio was not significantly correlated with CBF. We have recently reported on high lactate and low CBF during the acute phase following SAH in patients who later developed DCI (26). Different studies report on different levels of CBF thresholds for ischemia. Previous studies using Xenon-CT have reported on cortical CBF in awake normal subjects to be 52 ± 10 ml/100 g/min (27). In an additional study, comatose patients following head injury were compared to normal subjects and CBF threshold of 55.3 ml/100 g/min was defined as hyperemia (28). Our recent report on patients suffering severe SAH showed that patients who later develop DCI have initial low CBF levels of 23.7 ml/100 g/min compared to 37.5 ml/100 g/min in those who do not develop DCI (26). Current results are in line with previous findings and emphasize the important role of lactate in correlation with CBF in patients suffering SAH.

It is recommended that in patients suffering SAH, the MD catheter, if possible, should be placed in the vascular territory at risk (5). However, at our department, the MD catheter is placed in the right frontal lobe if there are no hematomas or infarction in conjunction to the ventriculostomy. This is based on the assumption that anterior brain a sensitive zone vascularized both by ACA and MCA, watershed areas, would offer an early warning signal of hypoperfusion and development of ischemia. In addition, this approach is logistically more feasible for the neurosurgeon on-call. In this study, we investigated how well the CBF in different vascular territories in the right hemisphere correlated with global CBF and if low CBF in different vascular territories was correlated to pathological findings from MD placed in right frontal lobe. We found a strong and significant correlation between global CBF and all three vascular territories in the right hemisphere. However, pathological values indicated by high lactate were correlated with ACA territory but not with MCA and PCA territories. This may be as expected since these territories are less covered by a catheter placed in the right frontal lobe.

The CBF measurements in this study were performed during day 0–3 following onset of SAH. Further studies are needed to evaluate the association between CBF and MD parameters at later time points after SAH with the catheter in right frontal lobe, given the increased risk of vasospasm and delayed focal cerebral ischemia. Another methodological limitation is the potential influence of artifacts from the EVD and MD catheter that may give inaccurately low CBF levels. This problem could not be avoided completely but is probably of minor magnitude. The artifacts were very small and comprised a minor proportion of the ROI volume analyzed.

A limitation of Xenon-CT CBF measurement compared to other methods such as PET is lower resolution and that only CBF can be quantified. However, PET is a complex and costly procedure with a need of cyclotron, while bedside Xenon-CT is more economical and accessible imaging technique with few adverse effects that can be used in the routine NIC to measure CBF.

In conclusion, the results of this study, using bedside Xenon-CT day 0–3 after SAH with simultaneous MD monitoring, show correspondence between high lactate levels and low regional CBF in the territory of right ACA but not in the middle and posterior vascular territories, when the MD catheter is placed in the right frontal lobe.

## Ethics Statement

The Uppsala University Regional Ethics Review Board for clinical research granted permission to undertake the study. Written informed consent was obtained from all patients or their proxy for study participation. The study was also approved by the local Radiation Safety Authority.

## Author Contributions

ER: design, data acquisition, analysis, and manuscript preparation. HE: Xenon-CT performance and manuscript preparation. TH, ER-E, and AL: data acquisition. PN: manuscript preparation. LH: data acquisition and manuscript preparation. PE: design and manuscript preparation.

## Conflict of Interest Statement

The authors declare that the research was conducted in the absence of any commercial or financial relationships that could be construed as a potential conflict of interest.
